# Are Posttraumatic Stress Symptoms and Avoidant Coping Inhibitory Factors? The Association Between Posttraumatic Growth and Quality of Life Among Low-Grade Gliomas Patients in China

**DOI:** 10.3389/fpsyg.2019.00330

**Published:** 2019-02-19

**Authors:** Junyi Li, Lijun Sun, Xiaoyu Wang, Cuicui Sun, Shupeng Heng, Xiangen Hu, Wei Chen, Fujun Liu

**Affiliations:** ^1^School of Psychology, Sichuan Normal University, Chengdu, China; ^2^School of Psychology, Xinxiang Medical University, Xinxiang, China; ^3^Department of Neurosurgery, West China Hospital, Sichuan University, Chengdu, China; ^4^School of Psychology, Central China Normal University, Wuhan, China; ^5^School of Psychology, Henan Normal University, Xinxiang, China

**Keywords:** low-grade gliomas, quality of life, posttraumatic growth, coping strategy, posttraumatic stress symptoms

## Abstract

**Background:** Diagnosing with low-grade gliomas (LGGs) can be a very shocking and stressful experience, a traumatic event potentially leading to the development of posttraumatic stress symptoms (PTSS), and posttraumatic growth (PTG). Understanding how patients cognitively and behaviorally response to their diagnosing is also important to postoperative treatment. Thus, the current study explored the association between PTG and quality of life (QoL) of Chinese patients with LGGs. The moderation effects of coping strategies and PTSS on the relationship between PTG and QoL have been examined as well.

**Methods:** Posttraumatic stress symptoms, Posttraumatic growth, coping strategies, and QoL were measured by using self-report surveys. Three hundred and thirty patients completed surveys approximately 1 month after surgery. We used three multiple regression models and added interaction terms in these models to test the moderation effects of PTSS and coping strategies on the relationship between PTG and QoL.

**Results:** The results of hierarchical multiple regression suggested that PTG significantly predicted QoL, both PTSS and coping strategies moderated the association between PTG and QoL. Specifically, the association between PTG and QoL for patients who have non-significant PTSS is stronger than those who have significant PTSS. Furthermore, as the score of Avoidant Coping increases, the association between PTG and QoL becomes weaker.

**Conclusion:** Posttraumatic growth may help to improve the QoL of LGGs patients, but PTSS and Avoidant Coping impeded the positive effect of PTG on QoL.

## Introduction

Every year in China, approximately 15,000 individuals are diagnosed with low-grade gliomas (LGGs) (Chinese Glioma Collaboration Group and Chinese Glioma Genome Atlas, 2004; Lu et al., 2012). LGGs arise from the glial matter in the brain, which includes all World Health Organization grade I and II gliomas. In some cases, LGGs will recur and progress to high-grade gliomas. Treating LGGs is particularly challenging due to the highly infiltrative nature making all treatment strategy palliative. Glimos patients have a poor prognosis, short survival time, and severe functional impairment (Chen, 2013). Thus, improving QoL in patients with LGGs is of utmost importance in the neurosurgical context. However, nurses and physicians working in oncological settings may not be sufficiently aware of the possible psychological reactions to the illness experienced by their patients (Casellas-Grau et al., 2018). Knowledge of psychological effect on QoL may be helpful for evaluating the prognosis of LGGs patients. It may also be useful when clinicians try to make treatment schedule to improve the QoL in LGGs patients. According to Wilson & Cleary Model of Health-related quality of life (QoL) (Wilson and Cleary, 1995; Bakas et al., 2012), biological and physiological variables, symptom status, functional status, and general health perceptions are associated with overall QoL. Furthermore, psychological factors (e.g., depression and posttraumatic stress symptoms) can have causal relationships with these variables at every level of the model (Wilson and Cleary, 1995). Based on this theoretical framework, this study emphasizes the influence of psychological factors on patients’ QoL, especially the influence of PTSS, PTG, and coping strategies.

Diagnosing with LGGs (or other cancers) can be a very shocking and stressful experience. According to DSM-IV criteria A (American Psychiatric Association, 2000), a traumatic event like diagnosing with cancers potentially leading to the development of PTSS and PTG. PTSD includes a persistent re-experiencing of the traumatic event, a persistent avoidance of stimuli associated with the event and the numbing of general responsiveness, as well as persistent symptoms of increased arousal. Symptoms must last at least 1 month and cause significant impairment in functioning. Many studies have found that diagnosing with cancer could trigger PTSS (Andrykowski et al., 1998; Al Jadili and Thabet, 2017). These symptoms will further negatively affect patients’ QoL (Ha et al., 2014).

While general research has traditionally focused on the adverse effects of trauma, positive outcomes such as PTG are also explored due to the influence of positive psychology. PTG refers to the positive psychological change that can occur as a result of a struggle with highly challenging adverse life events (Tedeschi and Calhoun, 1996). Campanella et al. (2017) found patients with LGGs appear to experience a deep psychological change and maturation, closely resembling PTG. Wang et al. (2018) also reported a positive association between PTG and QoL among LGGs patients. These results suggested that PTG may potentially improve the QoL for LGGs patients. However, results from studies that focused on cancers are contradictory. Some studies reported a positive relation between PTG and QoL (Harrington et al., 2008), but a few studies also found null or negative relations between them (Lechner et al., 2006; Stanton et al., 2006). Some possible factors may explain these disparities. First, different cancer may have a disparate impact on the relationship between PTG and QoL, differences in the methods of sampling and research design may also contribute these disparities; thus, it is necessary to examine the relationship between PTG and QoL under particular cancer (e.g., LGGs). Second, some potential variables may moderate the relationship between PTG and QoL, which makes this issue more complex. Therefore, it would be intriguing to discover if there are some enhancing or inhibitory factors will make the relationship between PTG and QoL becomes stronger or weaker.

The previous study found that PTG had a buffering effect on the relationship between PTSD and QoL among breast cancer survivors (Morrill et al., 2008). Kershaw et al. (2004) reported active coping strategies was associated with higher QoL and avoidant coping was associated with lower QoL; the negative relationship between avoidant coping and QoL was strongest when patients had low levels of symptom distress (Kershaw et al., 2004). These studies suggested coping strategies and PTSS may play essential roles in the relationship between PTG and QoL. Ehlers and Steil (1995) appraisal model of depression indicated intrusive cognition and intrusive memories (indices of unsuccessful emotional processing of the trauma) are particularly associated with PTSS and represents a core diagnostic feature of the disorder, which will further trigger depressive symptoms and then influence patients’ QoL (Williams and Moulds, 2008; Zhu et al., 2018), suggesting PTSS may have inhibitory effect on the relationship between PTG and QoL. Coping strategies are defined as a cognitive or behavioral response to something appraised as stressful and is a complex process that depends both on personality dispositions and environmental demands (Garrido-Hernansaiz et al., 2017). Gustafsson et al. (2006) investigated the coping strategies among LGGs patients; they found patients who used coping by escape-avoidance experience a high level of emotional distress. Avoidant strategies, such as behavioral disengagement and denial, may interfere with patients’ ability to problem-solve (related to QoL) or self-reflection (related to PTG) in the face of LGGs, whereas alcohol/drug use may create additional stress within the family that drains emotional and financial resources (related to QoL). Venting, as an avoidant coping strategy, refers to expressing negative feelings or letting unpleasant feelings escape, may interfere with family communication (Kershaw et al., 2004). Thus, we may hypothesize that avoidant coping style would inhibit the positive association between PTG and QoL. Researchers also proposed that multidisciplinary teams are needed for assessment and treatment of the different problems in patients with LGGs (Gustafsson et al., 2006). Therefore, the present study investigated the psychological features of LGGs patients and their relationships with QoL; especially we attempted to explore the moderation effects of PTSS and coping strategies on the relationship between PTG and QoL. The following hypotheses were proposed.

(1). PTG would positively predict QoL of LGGs patients in China.

(2). Posttraumatic stress symptoms would inhibit the positive relationship between PTG and QoL in LGGs patients.

(3). Avoidant coping strategies would inhibit the positive relationship between PTG and QoL in LGGs patients.

## Materials and Methods

### Participants

Before we selected participants, ethics approval was granted by the West China Hospital Sichuan University Institutional Human Research Ethics Committees. All patients have signed the consent form before study inclusion. Patients completed the questionnaires aided by the investigators at the hospital approximately 1 month after the surgery. Patients with histologically proven supratentorial LGG enrolled at the West China Hospital Sichuan University from February 2011 to July 2016. The inclusive criteria were as follows: (1) histologically confirmed diagnoses of WHO grade I or II glioma; (2) 18 years of age or older; (3) Karnofsy Performance Scale (KPS) > 60. The exclusion criteria were: (1) abnormal cognition mini-mental state examination (MMSE) < = 24; and (2) unable to read or understand questionnaire. Three hundred thirty-five patients enrolled and filled out the surveys. Five of them did not fully understand the surveys and were excluded.

### Measures

#### Demographic and Medical Variables

Demographics and medical features were obtained from hospital records, including gender, age, occupation, education, marital status, medical insurance, tumor grade, tumor position, excision, therapy method, and complication. We estimated socioeconomic status (SES) score for each participant according to their occupation (Li, 2005).

#### Mini-Mental State Examination (MMSE)

Mini-mental state examination was used to screen the participants in our study, which is one of the most widely used screening tests in epidemiological studies. The MMSE consists of a variety of items, has a maximum score of 30 points. The items have been grouped into seven categories, each rationally representing a different cognitive domain or function: orientation to time; orientation to place; registration of three words; attention and calculation; recall of three words; language and visual construction (Tombaugh and McIntyre, 1992). MMSE scores are frequently used to classify the severity of cognitive impairment into three levels: severe cognitive impairment (0–17), mild cognitive impairment (18–23), no cognitive impairment (24–30).

#### PTSD Checklist-Civilian Version (PCL-C)

Posttraumatic stress disorder symptoms were measured by using PCL-C, which is a 17-item questionnaire that assesses criteria B, C, and D of the PTSD construct consistent with the DSM-IV. Participants were asked how often they had been bothered by each symptom in the past month on a 1 (not at all) to 5 (extremely) Likert-type scale, for a range of 17–85. Scores of 38 for women and 44 for men was used as a cutoff to provide descriptive information about the sample regarding the proportion who screened positive for a likely diagnosis of PTSD (Kearney et al., 2012). The internal consistency of PCL-C total score was 0.71∼0.88.

#### Brief COPE Scale

Coping strategies were measured with the Brief COPE scale, which is a shorter version of the original 60-item COPE scale developed by Carver (1997). This version of the Brief COPE scale assessed twelve coping strategies, including self-distraction, active coping, denial, alcohol/drug use, use of emotional support, behavioral disengagement, venting, positive reframing, planning, use of humor, acceptance, and religion. Each strategy includes two items. Patients were asked how much they used different coping strategies within the past month. In the present study, we only measured nine coping strategies, since religion strategy was overlapping with the spiritual change in posttraumatic growth (PTG). In short, the coping strategies used in the current study were same with Kershaw et al. (2004)’s study. All coping strategies were classified into two types: active coping and avoidant coping (Kershaw et al., 2004). Denial, alcohol/drug use, behavioral disengagement, and venting belongs to avoidant coping. Use of emotional support, positive reframing, active coping, planning, and acceptance are viewed as active coping. Response options in this scale range from 0 (I haven’t been doing this at all) to 3 (I have been doing this a lot). The internal consistency of active coping and avoidant coping were 0.74 and 0.83, respectively.

#### Posttraumatic Growth Inventory (PTGI)

Posttraumatic growth was measured by using the Chinese version of PTGI, which is an instrument for assessing the positive change of persons who experienced traumatic events (Chen et al., 2012). The PTGI measures five different dimensions of life, such as relationships to others, new possibilities, personal strength, spirituality, and appreciation of life. Participants were asked to report the degree to certain changes occurred in their life as a result of their crisis on a six-point Likert scale. The response format ranged from 0 (I did not experience this change as a result of my crisis) to 5 (I experienced this change to a very great degree as a result of my crisis). The Chinese version of PTGI has good internal consistency, ranged from 0.66∼0.85 (Chen et al., 2012). The internal consistency in the present study was 0.70∼0.83.

#### The Functional Assessment of Cancer Therapy-Brain (FACT-Br)

Functional assessment of cancer therapy-brain was used to measure the general QoL. QoL scale consists of five dimensions: physical well-being (7 items), social well-being (7 items), emotional well-being (6 items), functional well-being (7 items), and concerns relevant to patients with brain tumors (23 items). Participants were asked to respond on a five-point Likert scale ranging from 0 (not at all) to 4 (very much). The internal consistency in the current sample was 0.63∼0.85.

### Data Analysis

We used R programming to preprocess data and fit linear models. Specifically, confirmatory factor analysis was conducted to ensure the construct validity of Brief COPE scale. Hierarchical linear models were fitted to test the moderation effects of PTSS and coping strategies on the relationship between PTG and QoL. We standardized continuous variables (e.g., PTG, SES, and QoL) and dummy coded categorical variables (e.g., PTSS and tumor position). Besides, variables that were significantly correlated with QoL (i.e., insurance, SES, tumor grade, seizure, PTG, PTSS, and avoidant coping) and interaction terms were included in the linear models.

## Results

### Missing Values

**FIGURE 1:**
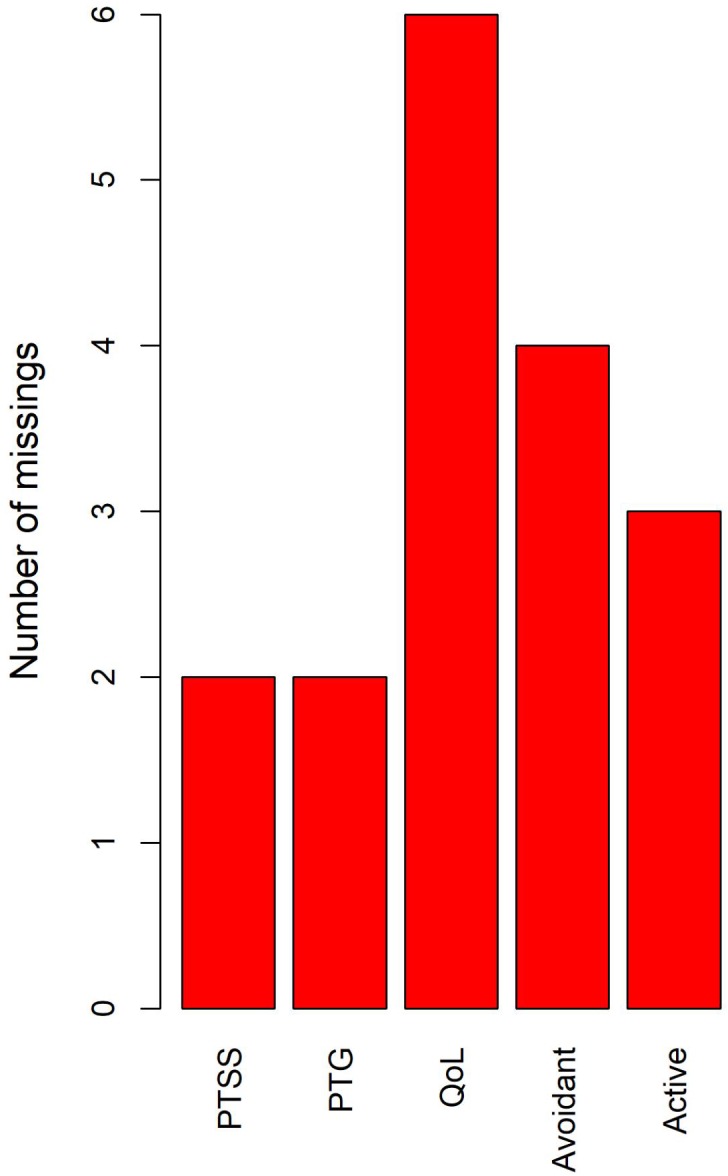
The missing values in the current study. The bar stands for the number of missing values in the corresponding variables, not the number of participants with missing values.

We found 18 missing values on the total score of the five scales (i.e., PCL-C, Brief COPE scale, PTGI, and FACT-Br). Specifically, 17 participants (5.15%) skipped one or two items in these scales (see Figure 1), which can be identified as true missing. This is probably because of carelessness. This missing pattern might be identified as MCAR (i.e., the probability of missing data on variable Y is unrelated to the true value of Y or other variables in the dataset). Thus, multiple imputations (mice package in R^1^) was used to fill the missing values in the dataset (Graham, 2009).

### Confirmatory Factor Analysis

**FIGURE 2:**
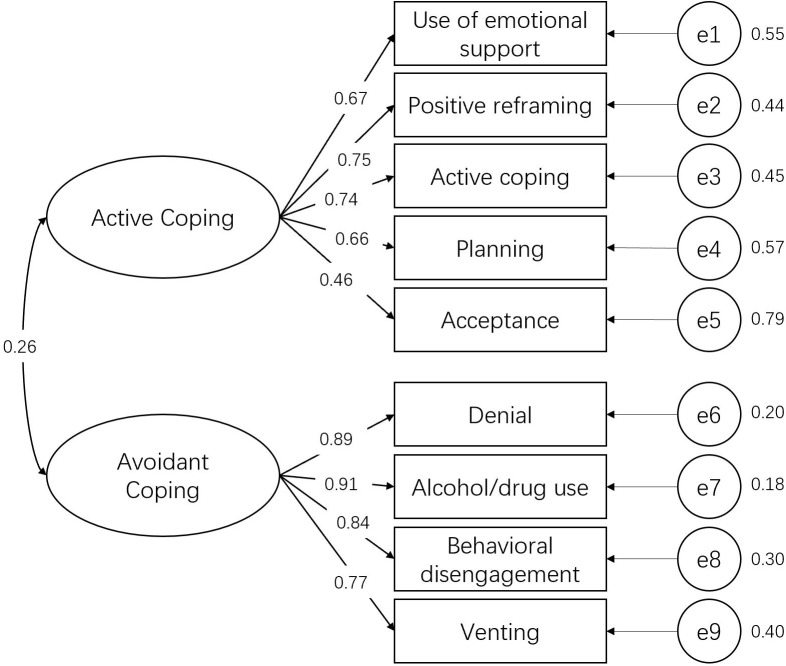
The result of confirmatory factor analysis.

The results showed that confirmatory factor analysis (lavaan package in R^2^) indicated the construct validity of Brief COPE scale was acceptable (see Figure 2). For the active coping, the standardized factor loadings of the five observed variables (i.e., emotional support, positive reframing, active coping, planning, and acceptance) on the latent variable (i.e., active coping) were 0.67 (*p* < 0.001), 0.75 (*p* < 0.001), 0.74 (*p* < 0.001), 0.66 (*p* < 0.001), and 0.46 (*p* < 0.001), respectively. As for the avoidant coping, the standardized factor loadings of the four observed variables (i.e., denial, alcohol/drug use, behavioral disengagement, and venting) on the latent variable (i.e., avoidant coping) were 0.89 (*p* < 0.001), 0.91 (*p* < 0.001), 0.84 (*p* < 0.001), and 0.77 (*p* < 0.001). The correlation between the two latent variables (i.e., avoidant coping and active coping) was 0.26. The model fit indices were: X^2^ = 135.37 (26), *p* < 0.001; CFI = 0.93; TLI = 0.90; NFI = 0.91; SRMR = 0.07; RMSEA = 0.10.

### Descriptive Statistics

As shown in Table 1, one hundred eighty-eight participants were male (57.00%) and 142 were female (43.00%) and the age range was from 18 to 67 years with a mean age of 40.20 (*SD* = 11.11). The majority of participants were married (70.00%). A total of 211 (63.90%) participants had medical insurance, and 119 (36.10%) without. Most participants live in the urban area (72.40%). Regarding tumor location, 150 (45.50%) of tumors were located in the left hemisphere, 172 (52.10%) were located in the right hemisphere. 8 (2.40%) tumors involved bilateral hemispheres. Among all the patients, 108 (32.70%) had the uncontrolled seizure. As for the PTSS, 179 (54.20%) patients’ PCL-C scores were greater than the cutoff (i.e., significant PTSS). In addition, we found the mean score of PTG and QoL was 52.63 (*SD* = 11.73) and 135.10 (*SD* = 15.69), respectively.

**Table 1:** Patient demographics and medical characteristics.

Demographics and medical characteristics	Sample size and proportion
Gender	
Male	188 (57.00%)
Female	142 (43.00%)
Marital status	
Single	84 (25.50%)
Married	231 (70.00%)
Divorce	6 (1.80%)
Widowed	5 (1.50%)
Others	4 (1.20%)
Medical insurance	
Yes	211 (63.90%)
No	119 (36.10%)
Residence	
Urban	239 (72.40%)
Rural	91 (27.60%)
Tumor grade	
1	121 (36.70%)
2	209 (63.30%)
Tumor position	
Left	150 (45.50%)
Right	172 (52.10%)
Both sides	8 (2.40%)
Excision	
Total	166 (50.30%)
Subtotal	141 (42.70%)
Biopsy	23 (7.00%)
Therapy	
Just surgery	128 (38.80%)
Surgery +Radiotherapy	176 (53.30%)
Surgery +Chemotherapy	10 (3.00%)
Surgery +Radio & Chemo	16 (4.80%)
PTSS	
Non-significant PTSS	151(45.80%)
Significant PTSS	179(54.20%)
Uncontrolled seizure	
Yes	108 (32.70%)
No	222 (67.30%)

Table 2 presented the Pearson’s correlation coefficients between QoL and the potential influence factors, including gender, residence, age, medical insurance, SES, complication, and tumor position, excision, adjuvant therapy, PTG, PTSS, and coping. The results suggested that QoL was significantly associated with medical insurance (*r* = 0.22, *p* < 0.001), SES (*r* = 0.25, *p* < 0.001), tumor grade (*r* = 0.13, *p* < 0.05), complication (*r* = −0.40, *p* < 0.001), PTG (*r* = 0.46, *p* < 0.001), PTSD (*r* = −0.45, *p* < 0.001), and avoidant coping (*r* = −0.19, *p* < 0.01).

**Table 2:** Correlations of study variables.

	1	2	3	4	5	6	7	8	9	10	11	12	13	14	15	16
1.Gender	1.00															
2.Age	0.12^∗^	1.00														
3.Marital status	0.11	0.31^∗∗∗^	1.00													
4.Insurance	0.00	0.13^∗^	−0.11^∗^	1.00												
5.Residence	−0.08	0.01	0.07	−0.07	1.00											
6.SES	0.05	−0.03	0.03	0.15^∗∗^	0.00	1.00										
7.Tumor grade	0.08	0.11^∗^	0.09	−0.13^∗^	−0.02	0.05	1.00									
8.Position	−0.08	0.11^∗^	0.01	0.10	0.06	−0.02	−0.04	1.00								
9.Excision	−0.07	0.15^∗∗^	0.06	0.09	0.00	0.06	0.13^∗^	0.16^∗∗^	1.00							
10.Adjuvant therapy	0.02	0.14^∗^	0.10	0.17^∗∗^	−0.07	0.06	0.32^∗∗∗^	0.12^∗^	0.41^∗∗∗^	1.00						
11.Seizure	−0.05	0.04	−0.02	−0.01	0.00	−0.03	0.02	0.10	0.06	0.12^∗^	1.00					
12.PTG	0.02	−0.01	−0.09	0.14^∗^	−0.09	0.12^∗^	−0.14^∗^	0.08	0.00	−0.01	−0.22^∗∗∗^	1.00				
13.QoL	−0.02	−0.07	−0.05	0.22^∗∗∗^	0.04	0.25^∗∗∗^	−0.13^∗^	0.10	−0.06	−0.10	−0.40^∗∗∗^	0.46^∗∗∗^	1.00			
14.PTSS	0.05	0.06	0.06	−0.14^∗∗^	−0.06	−0.19^∗∗^	0.15^∗∗^	−0.05	0.01	0.09	0.04	−0.15^∗∗^	−0.45^∗∗∗^	1.00		
15.Avoidant coping	0.06	−0.03	0.02	−0.08	−0.05	−0.11^∗^	−0.01	−0.02	0.08	0.05	0.00	0.17^∗∗^	−0.19^∗∗^	0.55^∗∗∗^	1.00	
16.Active coping	0.01	−0.06	−0.12^∗^	−0.01	−0.07^∗^	0.06	0.03	−0.01	−0.02	−0.04	−0.06	0.34^∗∗∗^	0.07	−0.05	0.11	1.00

*^∗^p < 0.05, ^∗∗^p < 0.01, ^∗∗∗^p < 0.001.*

### Hierarchical Regression Analysis

Hierarchical regression analysis was performed to analyze the association of PTG on QoL. Considering that the demographics and medical features might associate with QoL, it is necessary to control them as confounding variables. Specifically, we fitted six linear models (i.e., two PTSS models, two active coping models, and two avoidant coping models) for predicting QoL. Components and interaction terms were included in the regression models in separate steps. Table 3 presented the results of the hierarchical regression analysis. We found medical insurance, SES, complication, PTG, PTSS, and avoidant coping significantly correlated with patients’ QoL at 1 month after the operation. The interaction terms in PTSS model, active coping model, and avoidant coping model were significant.

**Table 3:** Standardized regression coefficients (*β*) of the predictors in different regression models.

Predictors	PTSS models				Active coping models				Avoidant coping models			
	Step 1		Step 2		Step 1		Step 2		Step 1		Step 2	
	β	*t*	β	*t*	β	*t*	β	*t*	β	*t*	β	*t*
Insurance	0.21	2.40^∗^	0.18	2.15^∗^	0.27	2.82^∗∗^	0.27	2.93^∗∗^	0.24	2.59^∗^	0.22	2.46^∗^
SES	0.12	2.80^∗∗^	0.11	2.71^∗∗^	0.18	4.06^∗∗∗^	0.18	4.00^∗∗∗^	0.15	3.49^∗∗∗^	0.14	3.27^∗∗^
Tumor grade	−0.07	−0.80	−0.05	−0.61	−0.14	−1.45	−0.13	−1.38	−0.14	−1.56	−0.10	−1.13
Seizure	−0.63	−7.17^∗∗∗^	−0.59	−6.78^∗∗∗^	−0.66	−6.83^∗∗∗^	−0.67	−6.98^∗∗∗^	−0.64	−6.89^∗∗∗^	−0.61	−6.56^∗∗∗^
PTG	0.29	6.88^∗∗∗^	0.43	6.94^∗∗∗^	0.38	7.64^∗∗∗^	0.35	7.07^∗∗∗^	0.39	8.62^∗∗∗^	0.40	8.90^∗∗∗^
PTSS	−0.71	−8.34^∗∗∗^	−0.71	−8.45^∗∗∗^								
PTG × PTSS			−0.25	−3.02^∗∗^								
Active coping					−0.09	−1.83	−0.08	−1.64				
PTG × Active coping							0.12	2.73^∗∗^				
Avoidant coping									−0.23	−5.22^∗∗∗^	−0.21	−4.74^∗∗∗^
PTG × Avoidant coping											−0.14	−3.15^∗∗^
_Δ_*Adjust R^2^*	0.01				0.01				0.02			
_Δ_*F*	9.11^∗∗^				7.43^∗∗^				9.90^∗∗^			

*PTSS is a categorical variable, it stands for whether the participant have significant PTSD symptoms. ^∗^p < 0.05, ^∗∗^p < 0.01, ^∗∗∗^p < 0.001.*

### Moderation Analysis

**FIGURE 3:**
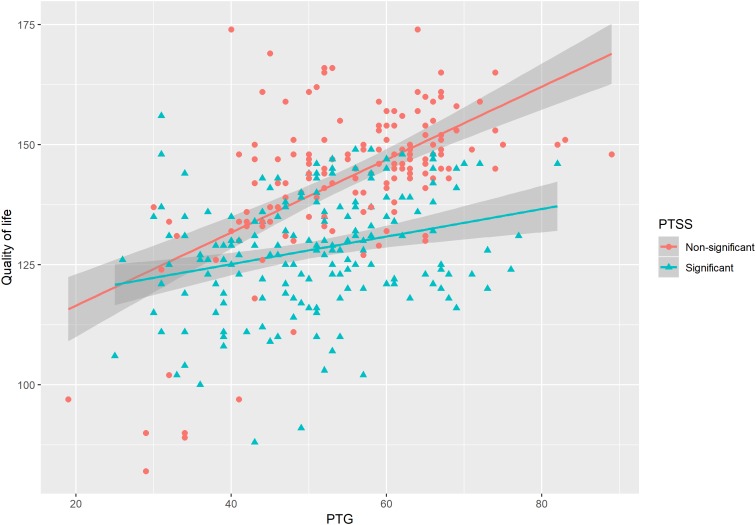
The moderating effect of PTSS on the relationship between posttraumatic growth (PTG) and QoL.

**FIGURE 4:**
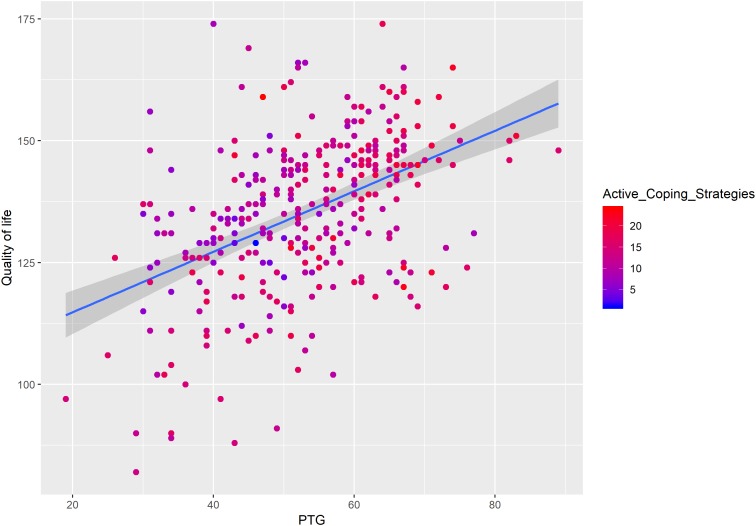
The moderating effect of active coping on the relationship between PTG and QoL.

**FIGURE 5:**
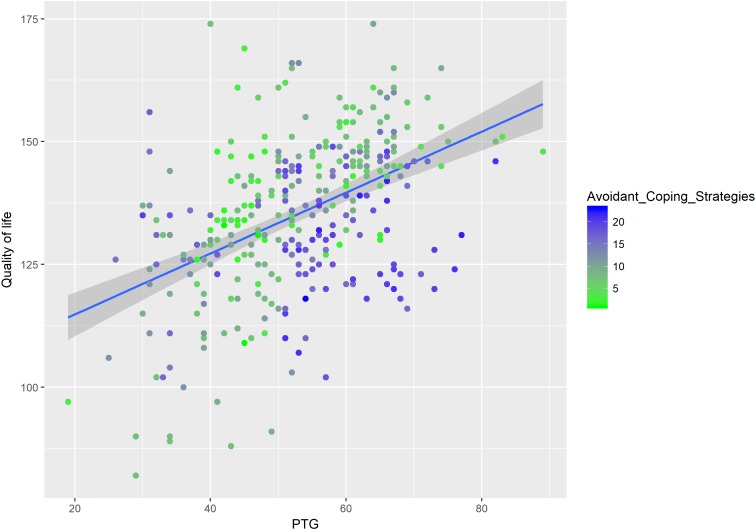
The moderating effect of avoidant coping on the relationship between PTG and QoL.

We added the interactions terms of PTG and PTSS, PTG and active coping, and PTG and avoidant coping in the models to test the moderation effects of PTSS and coping strategies on the association between PTG and QoL. As shown in Table 3, the interaction term of PTG and PTSS had a significant negative association on QoL (β = −0.25, *t* = −3.02, *p* < 0.01), which means PTSS had an inhibitory effect on the relationship between PTG and QoL (see Figure 3). We also found the interaction term of active coping and PTG predicted QoL (β = 0.12, *t* = 2.73, *p* < 0.01, Figure 4). As for the interaction term of avoidant coping, as the score of avoidant coping increase, the relationship between PTG and QoL become weaker (β = −0.14, *t* = −3.15, *p* < 0.01, Figure 5). Therefore, the moderation effects of PTSS and coping strategies were found.

## Discussion

In the current research, we investigated 330 patients with LGGs at approximately 1 month after the surgery. PTG, PTSS, coping strategies and QoL were analyzed using hierarchical regression and moderation effect model. Results indicated that PTG significantly predicted QoL, PTSS and coping strategies moderated the relationship between PTG and QoL. In this section, we discussed the implications of these findings, the limitations of the present study, and future research directions.

### The Effect of PTG on QoL of LGGs Survivors

We found PTG could significantly predict QoL after controlling the effects of medical features and demographics, which suggested patients who had a higher level of PTG may experience better QoL. Hypothesis 1 was verified. This result was consistent with previous findings. For example, Holtmaat et al. (2017) found the association between higher PTG with better social functioning. Casellas-Grau et al. (2018) reviewed 72 articles focused on cancer; they reported PTG resulted inversely associated with depressive and anxious symptoms and directly related to hope, optimism, spirituality and meaning. In a longitudinal study, Tanyi et al. (2014) found a significant positive correlation between social/family well-being and PTG. The affective-cognitive processing model of PTG may help to interpret how PTG affect QoL (Joseph et al., 2012). Diagnoses of cancer modified patients’ pre-existing assumptions about themselves and the world. After a series of reflective pondering, the assumptive world of the patients was modified in light of the new trauma-related information (e.g., diagnoses of LGGs). This accommodation process may help LGGs patients experience a deep psychological change and maturation, which contribute to the increase of QoL. Therefore, the evaluation of PTG in LGGs patients is worthy, since it may promote a better adaption to the illness.

### The Inhibitory Effect of PTSS on the Relationship Between PTG and QoL

Posttraumatic stress symptoms had a significant moderation effect on the relationship between PTG and QoL, which accorded with our hypothesis 2. Specifically, when patients have significant PTSS, the relationship between PTG and QoL become weaker (see Figure 3). Many studies have evaluated the prevalence, predictors, and correlates of cancer-related PTSS and diagnoses (Cordova et al., 2017). A substantial proportion of people with cancer might be experience their diagnosis and treatment as traumatic. Gold et al. (2012) investigated 289 adult oncology patients and found that 45% of the sample met the diagnostic criteria for PTSD and partial PTSD. In their study, Hahn et al. (2015) found 29% of the cancer survivors had PTSS. Thus, accessing PTSS and examining how it affects PTG and QoL are important to patients. In fact, the association between PTSS and QoL have been documented by previous studies. For example, Jung et al. (2017) found the higher level of PTSS were negatively related with QoL. Evidence from meta-analysis also suggested cancer-related PTSS were negatively correlated with QoL (Shand et al., 2015). But how PTSS moderated the association between PTG and QoL? A theoretical framework of PTG posits that traumatic event can shatter assumptions about the self and the world (Joseph and Linley, 2005, 2006), which triggers automatic ruminative activity. For some patients (e.g., the patients who have significant PTSD), this ruminative activity was experienced as posttraumatic stress symptoms (Shand et al., 2015), which would negatively affect QoL. However, for the patients who have non-significant PTSD or engage in active coping strategies (e.g., positive reappraisal), there may be deliberate rumination characterized by narrative development and search for meaning (Kangas et al., 2002; Linley and Joseph, 2005). This meaning-making process would help to improve the QoL of LGGs patients. Taken together, PTSS may inhibit the positive effect of PTG on QoL.

### The Inhibitory Effect of Avoidant Coping on the Relationship Between PTG and QoL

Besides the inhibitory effect PTSS, the present study found the inhibitory effect of avoidant coping on the relationship between PTG and QoL (see Figure 5). This result supported hypothesis 3. The enhancing effect of active coping was also found; as the score of active coping increased, the relationship between PTG and QoL became stronger (see Figure 4). Coping has two basic functions, either to manage or alter the problems causing distress or to regulate the emotions caused by these problems. Thus, adopting appropriate coping strategies may be helpful to LGGs patients. Some studies showed that LGGs patients may adopt some avoidant coping strategies, which needs clinicians to pay attention. For example, Edvardsson and Ahlström (2005) found LGGs patients adopt several coping strategies, including searching for a solution, refraining from and avoiding, laughing and joking, and caring about self. Gustafsson et al. (2006) reported emotion-focused coping is a dominated strategy in their sample, the patients with LGGs more often tried to change the significant of the situation or tried to escape mentally; this coping had a significant association with lower level of emotional functioning. Among breast cancer patients, Holland and Holahan (2003) found active coping strategies were positively correlated to psychological well-being and positive health behaviors, whereas avoidant coping was negatively related to psychological well-being. Similar results were also reported in Kershaw et al. (2004) study. Again, the theoretical framework of PTG may help to interpret the moderating effect of coping strategies (Joseph and Linley, 2006). Traumatic event (e.g., LGGs) would shatter patients’ assumptions about themselves and the world and trigger ruminative activity. For patients who adopt avoidant coping strategies (e.g., negative appraisals and cognition avoidance), they are more likely to experience greater distress, the distress, in turn, may affirm the patients’ negative appraisals and lead to an additional increase in levels of distress (Williams and Moulds, 2008). This psychological process would reduce the QoL of LGGs patients. However, for patients who adopt active coping strategies, they are more likely to experience effective development of PTG (e.g., religious coping and positive reframing consistently showed significant association with PTG, Rajandram et al., 2011), which may further affect QoL.

## Limitations and Directions for Future Research

Some limitations of the present study should be noted. The first limitation was the use of self-report instruments to detect PTSS rather than the ratings of a clinician or the interview method. Thus, the self-reporting of PTSS might be considered less accurate. Second, the Cronbach’s α of emotional well-being dimension in FACT-Br was low, it should be cautious when generalize this result to other contexts. Third, the present study is a cross-sectional study, which means it is not possible to draw causal relationships between PTG and QoL. Third, the patients in the present study might not be fully representative of the greater patient population with LGGs, because patients with cognitive deficits were excluded. Fourth, it should be noticed that one strategy is not necessarily good or bad; rather it depends on the situation and the outcome that follows its particular use.

Despite these limitations, the current study had a relatively large sample size, which guaranteed enough statistical power. Considering clinicians working in the neurosurgical context may not be sufficiently aware of patients’ possible psychological reactions to LGGs, the current study provided evidence that PTG, PTSS, and coping style are important psychological processes to QoL of LGGs patients, which confirms Wilson & Cleary Model of Health-related QoL. This paper also helps to solve the disparities in the previous studies on the relationship between PTG and QoL among cancer patients by exploring the moderation effects of PTSS and coping strategies. Information like these is useful for clinicians when they try to make treatment schedule to improve the QoL in LGGs patients. It is worthy to evaluate PTG of LGGs patients and pay particular attention to those who have a higher level of PTSS and adopt avoidant coping strategies. Thus, manifold interventions in the psychosocial area during long-term cancer survival are required (Gustafsson et al., 2006). Future studies should continue to explore the potential mediators and moderators for the relationship between PTG and QoL so that we can have a deeper understanding of the crucial mechanism linking PTG and QoL and the boundaries of PTG effects. Support programs tailored to the different medical, physical and psychosocial needs of LGGs patients should be designed in the future to ensure the quality of care. Intervention studies could also be conducted to buffer PTSS and teach the appropriate coping strategies to LGGs patients.

## Conclusion

Posttraumatic growth has a positive association on QoL among patients with LGGs. However, this positive effect was inhibited by significant PTSS and avoidant coping strategies. Hence, interventions could be used to buffer PTSS and encourage patients to adopt active coping strategies.

## Author Contributions

JL conceptualized the research idea, performed the data analysis, and wrote the manuscript. XW collected the data, verified the results of statistical analysis, and supervised the whole research process. CS performed the data analysis and revised the manuscript. LS performed the data cleaning and revised the manuscript. SH revised the manuscript. XH provided the statistical support and revised the manuscript. WC collected the data and performed the raw data cleaning. FL collected the data and performed raw data cleaning.

## Conflict of Interest Statement

The authors declare that the research was conducted in the absence of any commercial or financial relationships that could be construed as a potential conflict of interest.

## Footnotes

1 https://cran.r-project.org/web/packages/mice/index.html

2 https://cran.r-project.org/web/packages/lavaan/index.html
